# Breast Tumor Identification in Ultrafast MRI Using Temporal and Spatial Information

**DOI:** 10.3390/cancers14082042

**Published:** 2022-04-18

**Authors:** Xueping Jing, Monique D. Dorrius, Mirjam Wielema, Paul E. Sijens, Matthijs Oudkerk, Peter van Ooijen

**Affiliations:** 1 Department of Radiation Oncology, University Medical Center Groningen, University of Groningen, 9700 RB Groningen, The Netherlands; x.jing@umcg.nl; 2 Department of Radiology, University Medical Center Groningen, University of Groningen, 9700 RB Groningen, The Netherlands; m.wielema@umcg.nl (M.W.); p.e.sijens@umcg.nl (P.E.S.); 3 Faculty of Medical Sciences, University of Groningen, 9700 RB Groningen, The Netherlands; m.oudkerk@umcg.nl; 4 Institute for Diagnostic Accuracy, 9713 GH Groningen, The Netherlands

**Keywords:** ultrafast breast MRI, lesion classification, deep learning

## Abstract

**Simple Summary:**

The diagnosis of breast cancer with MRI is based on both morphological evaluation and kinetic curve assessment. Current computer-aided diagnosis methods for malignancy determination mainly focus on morphology features but ignored the temporal information in dynamic contrast-enhanced MRI sequences. Malignant and benign lesions usually have different enhancement patterns during the wash-in phase. Ultrafast breast MRI with high temporal resolution can capture the inflow of contrast in breast lesions. This advantage of ultrafast MRI enables the combination of both temporal and spatial information for automatic breast lesion analysis model development. We found that temporal information helps to significantly improve the performance of breast lesion classification. This suggests that ultrafast MRI provides useful information for malignancy identification and temporal information, which is indispensable for similar model development.

**Abstract:**

Purpose: To investigate the feasibility of using deep learning methods to differentiate benign from malignant breast lesions in ultrafast MRI with both temporal and spatial information. Methods: A total of 173 single breasts of 122 women (151 examinations) with lesions above 5 mm were retrospectively included. A total of 109 out of 173 lesions were benign. Maximum intensity projection (MIP) images were generated from each of the 14 contrast-enhanced T1-weighted acquisitions in the ultrafast MRI scan. A 2D convolutional neural network (CNN) and a long short-term memory (LSTM) network were employed to extract morphological and temporal features, respectively. The 2D CNN model was trained with the MIPs from the last four acquisitions to ensure the visibility of the lesions, while the LSTM model took MIPs of an entire scan as input. The performance of each model and their combination were evaluated with 100-times repeated stratified four-fold cross-validation. Those models were then compared with models developed with standard DCE-MRI which followed the same data split. Results: In the differentiation between benign and malignant lesions, the ultrafast MRI-based 2D CNN achieved a mean AUC of 0.81 ± 0.06, and the LSTM network achieved a mean AUC of 0.78 ± 0.07; their combination showed a mean AUC of 0.83 ± 0.06 in the cross-validation. The mean AUC values were significantly higher for ultrafast MRI-based models than standard DCE-MRI-based models. Conclusion: Deep learning models developed with ultrafast breast MRI achieved higher performances than standard DCE-MRI for malignancy discrimination. The improved AUC values of the combined models indicate an added value of temporal information extracted by the LSTM model in breast lesion characterization.

## 1. Introduction

Dynamic contrast-enhanced magnetic resonance imaging (DCE-MRI) has a higher sensitivity compared with other modalities for breast cancer detection [1]. It is widely used for breast cancer staging, screening in high-risk women, and chemotherapy evaluation. For breast cancer screening, recent research reveals that supplemental MRI screening in a high-risk population, especially in women with extremely dense breasts, could significantly reduce the interval cancer rate compared with mammography alone [2]. For women with a lifetime breast cancer risk above 20%, annual MRI screening helps to detect cancers at an earlier stage than mammography [3]. However, the advantages of breast MRI are accompanied by higher cost and a higher false positive rate. 

During DCE-MRI scanning, the concentration of gadolinium contrast material in the permeable blood vessels of tumor tissue causes a decrease in the relaxation time of water protons and results in a high signal in T1-weighted images. The current DCE-MRI protocol usually contains multiple T1-weighted acquisitions after contrast. The diagnosis of breast lesions is highly dependent on the high spatial resolution and kinetic pattern in the wash-out phase, making MRI scanning tedious. To enable a wider use of breast MRI, protocols should be shorter to improve cost effectiveness [4,5]. Different abbreviated protocols have been proposed to shorten the scanning time [6,7,8]. Studies also showed that the diagnostic performance of abbreviated protocols matches the full diagnostic breast MRI protocol [9,10,11,12,13]. 

**Figure 1:**
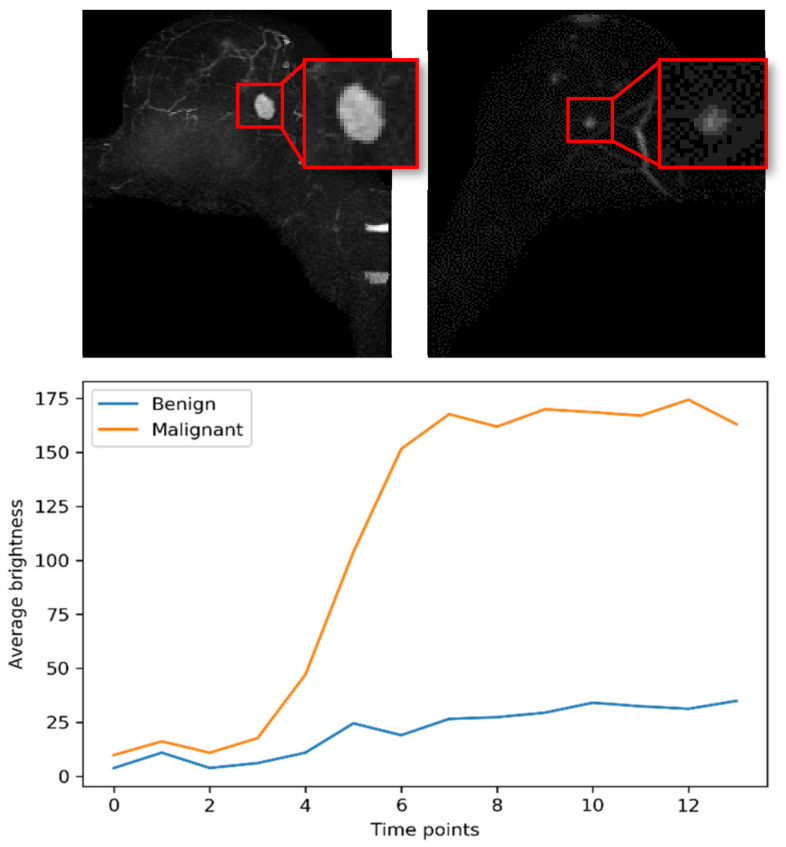
Maximum intensity projection images (MIP) of a malignant (**left**) and a benign (**right**) lesion and their enhancement curves during ultrafast MRI. Plotted curves are the average brightness of 10 × 10 pixels in the center of each lesion.

Most abbreviated protocols are based on T1-weighted imaging before and after contrast agent administration. T1-weighted acquisitions may be performed in different time resolutions. Conventional T1-weighted MRI has high spatial resolution, but is time consuming, while the time-resolved angiography with stochastic trajectories (TWIST) technique enables ultrafast acquisition at the cost of a lower spatial resolution [14,15]. Compared with the use of only one high spatial resolution post-contrast T1-weighted series for image interpretation [7], repeated acquisition of a T1-weighted sequence in TWIST enables not only morphological evaluation but also pharmacokinetic analysis of the wash-in phase (Figure 1). The current literature recognizes the critical role of kinetic analysis in the differentiation of benign and malignant lesions in ultrafast MRI. The ultrafast T1-weighted sequence might change the future of breast DCE-MRI and has proven useful for the characterization of breast lesions. Abe et al. [16] concluded that there was significant difference between benign and malignant lesions in enhancement rate and kinetic area under the curve (AUC) in ultrafast MRI, and that the differential utility of ultrafast imaging is comparable to standard kinetic assessment. Other studies [17] found that features (time to enhancement and maximum slope) derived from ultrafast MRI during initial enhancement could help improve the performance of differentiating between malignant and benign lesions, especially in case of non-mass enhancement. These studies revealed the potential of kinetic analysis in ultrafast MRI for breast lesions [7,17,18,19]. 

Recently, artificial intelligence (AI) was introduced to help discriminate benign from malignant lesions in breast MRI [20,21]. Most AI studies focus on 2D feature extraction with, either, radiomics methods [22,23] or convolutional neural networks (CNN) [24]. Even though it has proven to be a crucial factor and is widely used in clinical practice, temporal information (kinetic features) has been ignored in the development of automated breast lesion analysis systems. Long short-term memory (LSTM) networks [25], which are widely used for time series analysis, have been employed for medical image analysis recently [26,27,28]. For example, Zou et al. [29] used an LSTM model for pharmacokinetic parameter estimation in head and neck cancer using a TWIST sequence and achieved promising results. These successful applications indicate that temporal information extracted by LSTM models may further improve the performance of automatic breast lesion diagnosis in DCE-MRI. 

We hypothesized that fully automatic deep learning-based methods, with only ultrafast DCE-MRI as input, could be used for malignancy identification. Therefore, the aim of this study is to evaluate the feasibility of this automatic deep learning-based method and investigate the usefulness of temporal information extracted with an LSTM model.

## 2. Materials and Methods

### 2.1. Study Population

This retrospective study was approved by the local Medical Ethics Review Board (METc Nr: 2018/652) with a waiver of the requirement for informed consent from each patient. A total of 1447 breast MRIs, including both screening and diagnostic examinations, from 809 patients who underwent breast MRI scans at our hospital between 2016 and 2019 were retrospectively included. Among the 1447 consecutive breast MRIs, 443 were excluded because no ultrafast DCE-MRI sequences had been acquired. Furthermore, 694 MRI scans from 374 patients with only focus/foci (enhancing dots with diameters smaller than 5 mm) or with no lesion reported were excluded. A total of 34 MRI scans for chemotherapy evaluation, 31 for breast implants, and 94 post-operative scans were also excluded, resulting in 151 MRI scans from 122 patients. For the remaining 151 MRI scans, left and right breasts were assessed separately for the presence of breast lesions. A total of 129 breasts with no suspicious lesion were excluded, and 173 breasts with one or more lesions were included in the final model development. 

**Figure 2:**
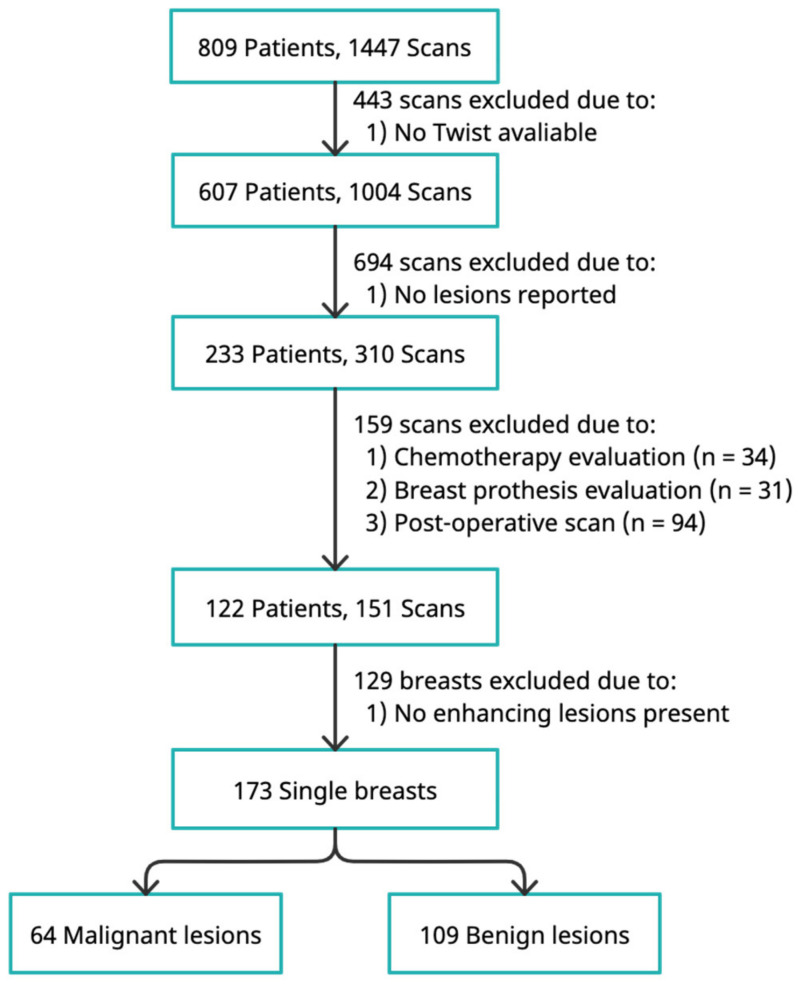
Flowchart of the patient inclusion.

The label of each breast was determined by the primary lesion contained. For breasts with no malignant lesions, the benign lesion with the biggest size was analyzed. For breasts with malignant lesions, the possible additional present benign lesions were not included in the analysis. The ground truth of those included lesions was obtained from pathology reports (surgery or core needle biopsy) or follow-up examinations (2 years follow up, verified by a senior radiologist). The final study dataset contained 109 benign and 64 malignant lesions (Figure 2).

### 2.2. MRI Protocol

All DCE-MRI scans were acquired on a 3 T (Siemens Magnetom Skyra, Siemens Medical Solutions, Erlangen, Germany) or 1.5 T (Siemens Magnetom Avanto_fit, Siemens Medical Solutions, Erlangen, Germany) scanner with a circularly polarized bilateral breast coil (Siemens). The protocol consisted of a pre-contrast T1 and T2 and the following sequences after contrast: DWI, TWIST, and four DCE-T1-weighted series. A measurement of 15 mL of contrast agent (Dotarem, Guerbet, Villepinte, France) was injected using a power injector. Acquisition parameters of the T1-weighted and TWIST sequence are list in Table 1.

### 2.3. Development of the Deep-Learning-Based Classification System

Breast segmentations were first performed by using a 3D U-Net [30] to remove the redundant background; the left and right breasts were then separated by splitting the breast regions in the middle. Maximum intensity projection (MIP) images were then generated by applying MIP operation on the subtraction volume of each sequence and the sequence before contrast agent injection. 

**Figure 3:**
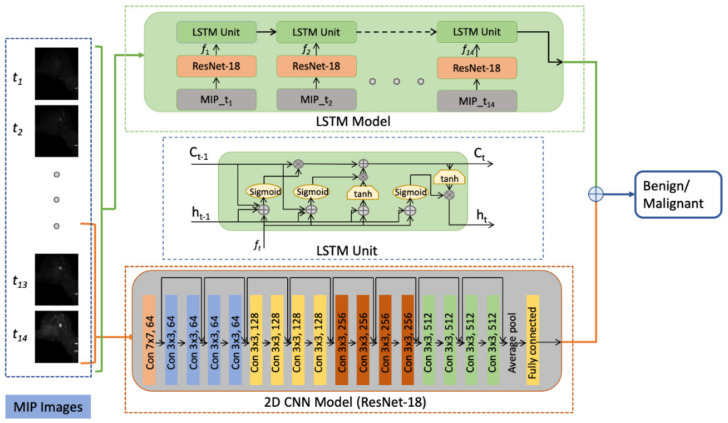
Pipeline of the proposed system. The 2D CNN model takes a maximum intensity projection (MIP) image of the last four acquisitions as input, while the LSTM model takes all 14 MIP images in a TWIST sequence as a single input. In the LSTM model, a RseNet-18 model was used for feature extraction. The extracted feature vector f_t_ was then inputted to the LSTM unit, in which C_t__-1_ represents the memory from last unit, h_t-1_ represents the output of the previous unit, C_t_ represents the memory of the current unit, and h_t_ represents the output of the current unit. The output probability of each model was added up to generate a combined prediction.

To take advantage of both morphological and temporal information provided by ultrafast breast MRI, a CNN model and a long short-term memory LSTM network were employed to extract morphological and temporal features, respectively. (Figure 3) The CNN model, modified from the pre-trained ResNet-18 [31] by replacing the last output layer, were trained with MIP images from the last four acquisitions in each ultrafast MRI series. The LSTM model, with the ResNet-18 model as feature extractor, was trained with the MIP clip (14 timed acquisitions of the ultrafast DCE sequence yielding 14 MIP images) of each ultrafast MRI series. For standard DCE-MRI, the same method was used for the training, where MIPs of the four T1-weighted acquisitions after contrast injection were used as the input for both the 2D CNN and LSTM model.

The output of each model was a numerical value between zero and one, which indicates the probability of malignancy. The performance of each model and their combination was evaluated with 100-times repeated stratified 4-fold cross-validation. During inference, the CNN models assessed each of the four MIP images from a single validation sample, and the highest output value was then determined as the malignancy probability of the sample. The output of the proposed system was then determined by averaging the predicted scores from both models. The receiver operating characteristic (ROC) curve was plotted for each model and the entire system based on the predicted probability. Sensitivity and specificity were used to evaluate the performance of each model with different thresholds applied.

Models were developed with Pytorch framework (v1.9.1) and trained with an NVIDIA Quadro P4000 GPU. For the 2D CNN (ResNet-18) model, transfer learning was adopted with pre-trained weights on ImageNet; convolution layers were frozen while only fully connected layers’ parameters were finetuned. The training was run for 30 epochs with a learning rate of 1e-4, a batch size of 16, and a momentum of 0.9. The Adam optimizer and a categorical cross-entropy loss function were used. For the LSTM model, a ResNet-18 model was used as feature extractor. The same hypermeters were used for training, except the batch size was reduced to four and training epochs were extended to 40. During training, MIP images were first resized to 224 × 224, and then sent into the models; random horizontal flip (probability of 0.5) and random rotation (within 10°) were performed for data augmentation. The training (30 epochs) and validation of the 2D CNN model took 28.8 and 1.5 min, respectively, while the training (40 epochs) and validation of the LSTM model took 34.3 and 2.2 min, respectively, in each fold.

## 3. Results

### 3.1. Patient and Lesion Characteristics

A total of 122 patients with 173 breasts were included in this study. The patient and lesion characteristics are provided in Table 2. The median age was 47 years (range, 24–80 years). The median size of all lesions was 13.0 mm (range, 5.0–110.0 mm), while for benign lesions it was 9.0 mm (range, 5.0–81.0 mm) and malignant lesions it was 22.0 mm (range, 6–110 mm).

### 3.2. Performance of Models

**Figure 4:**
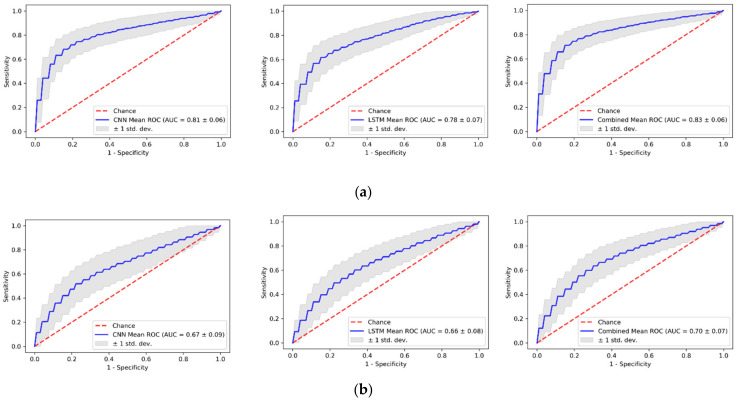
Receiver operating characteristic (ROC) curves for CNN, LSTM model, and their combination in the (**a**) TWIST and (**b**) standard DCE-MRI validation sets. The plots show the mean ROC of each model over 100-times repeated cross-validation, reflecting the variance of the curves when the dataset is split into different training and validation sets.

The ROC curves of each model for malignancy differentiation with TWIST sequences are shown in Figure 4. During the repeated stratified cross-validation, the 2D CNN showed a mean AUC of 0.81 ± 0.06, the LSTM network showed a mean AUC of 0.78 ± 0.07, while their combination showed a mean AUC of 0.83 ± 0.06. For standard DCE-MRI, the 2D CNN model showed a mean AUC of 0.67 ± 0.09, the LSTM network showed a mean AUC 0f 0.66 ± 0.08, and their combination showed a mean AUC of 0.70 ± 0.07.

The 2D CNN achieved a higher mean AUC than LSTM (*p* < 0.01), but the combined prediction could further improve the performance (all *p* < 0.01). There is clearly an added value in using the temporal information extracted by the LSTM model. A detailed analysis of the diagnosis performance with different cutoff values is listed in Table 3.

**Figure 5:**
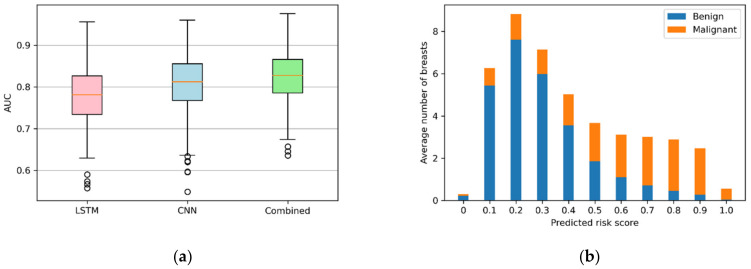
The boxplots of the mean AUC values of each model on the TWIST validation set (**a**) and the distribution of AI risk scores predicted by the combined model for benign and malignant lesions (**b**).

To better illustrate the classification patten of the models developed with ultrafast MRI, boxplots of the mean AUC values of each model during cross-validation are illustrated in Figure 5a. Compared with the 2D CNN and LSTM model, less dispersed AUC values and fewer outliers indicate an improved performance of the combination. The distribution of predicted risk scores of the combined model in relation to malignancy is shown in Figure 5b. Malignant lesions were shown to likely be predicted with a higher risk score compared with benign lesions. The mean risk scores for malignant and benign lesions were 0.588 (95% CI: 0.582, 0.594) and 0.294 (95% CI: 0.291, 0.297), respectively.

## 4. Discussion

In this study, we investigated the possibility of using a deep learning model for breast lesion discrimination with only ultrafast MRI as input. Motivated by recently discovered kinetic features derived from ultrafast MRI, we looked at the effectiveness of deep learning-based automatic pharmacokinetic analysis of the wash-in phase and possible improvements to morphological analysis. For this purpose, a 2D CNN model and an LSTM model were first trained and evaluated separately for the classification of benign and malignant lesions, and then combined by the fusion of the predicted scores of each model. 

The results showed that deep learning models could achieve high performance for lesion differentiation with only ultrafast breast MRI. The models developed with ultrafast MRI had a significantly higher mean AUC values compared with the model developed with standard DCE-MRI. Meanwhile, the combined model achieved the highest mean AUC value during repeated straited cross-validation. Even though the mean AUC value of the LSTM model was lower than the 2D CNN model, after integrating them together, the temporal information extracted by the LSTM model turned out to have added value for diagnostic performance. This upward trend was observed with both ultrafast and standard MRI.

Machine learning was also adopted for malignancy prediction in previous studies with different sequences. Platel et al. [32] used 5 morphological features and 18 uptake-curve features extracted from ultrafast MRI to train support vector machine (SVM) models. The AUC of the SVM models trained with only kinetic features or only morphological features were 0.80 and 0.79, respectively, while the SVM model trained with all the 23 handcraft features together showed an AUC of 0.85. These results are consistent with our findings, indicating that both temporal and morphological features are indispensable for the development of breast lesion classification models in ultrafast MRI. In another study, Dalmış et al. [24] developed a deep-learning-based classifier which takes image patches from TWIST, T2w, DWI sequences, and patient information as input. When only image patches were used, an AUC of 0.83 was achieved, while with combining all imaging and patient information, the AUC was 0.85. Pötsch et al. [22] extracted 86 pharmacokinetic enhancement features from TWIST sequences to train a multilayer perceptron (MLP) artificial neural network. The MLP model achieved an AUC of 0.83 on the test dataset and could help avoid 14.5% biopsies without false negatives. Radiomics has also been used for breast lesion differentiation [33,34,35].

Compared with previous studies developing classifiers with handcraft or radiomics features, in this work, we used a 2D CNN model and an LSTM model to automatically extract morphological and kinetic features separately. Instead of highly depending on the manually delineated lesion area for feature extraction, a big advantage of the proposed method is that neither manual annotation nor dedicated segmentation of the lesions are required before analysis. The models take raw images as input and provide predictions based on the content only; therefore, it could be easily integrated in the clinic workflow and works fully automated. 

What should also be noticed is that there is no associate relationship of identification ability between machine learning models and radiologists. Radiologists still rely more on high-resolution T1-weighted sequences for malignancy discrimination, which intuitively contradicts the finds of this paper. A possible explanation for the inferior performance of the standard DCE-MRI-based models might be that temporal analysis of the TWIST sequence with the LSTM model made up for the defect of morphological analysis caused by insufficient spatial resolution.

Despite these promising results achieved with ultrafast MRI sequences, we can easily envision the improvements when taking other sequences into consideration. For accurate diagnosis, delayed sequences are indispensable in clinical practice, especially the wash-out phase in DCE-MRI which provides irreplaceable functionality of indicating malignancy. However, in the trend of shortening MRI protocol for screening, identifying the malignancy of a tumor from an easily accessible sequence with a fully automatic model could provide support for a flexible scanning strategy, in which additional MRI sequences could be added from the protocol as needed. The flexibility of MRI could save time while maintaining the performance of breast cancer screening.

There are still several limitations of our study. This retrospective study had a relatively small dataset from a single medical center, and not all the benign lesions were pathologically confirmed. Even though the dataset has been randomly split 100 times to illustrate the robustness of the models, a large multivendor multicenter dataset is still needed for further validation. Another limitation of the work is that the study was conducted in a high-risk screening population and included pre-operation examinations; therefore, the prevalence of malignant lesions in our study is higher compared with the general screening population, which may weaken the model’s generalizability. Additionally, patient information has been proven effective to improve the decision-making of classification models, and properly integrating patient information may further improve the performance. Furthermore, we focused on the validation of the usefulness of temporal information extracted with an LSTM model; all the models were trained with the same hyperparameter setting and training strategy, which may lead to an underestimated performance of the system. Finally, models were trained and validated with the output of the primary lesion in each breast. Therefore, the predictions only revealed the status of primary lesions but overlooked associated lesions. Object detection models, which could locate each single lesion, may help address this problem.

## 5. Conclusions

In conclusion, deep learning models achieved promising results for the fully automatic differentiation of benign and malignant lesions. Kinetic information extracted by LSTM model has added value in the 2D CNN-based morphology analysis.

## Figures and Tables

**Table 1:** Acquisition Parameters for ultrafast and standard DCE-MRI.

Parameter	TWIST		T1-Weighted	
	1.5 T	3.0 T	1.5 T	3.0 T
TR/TE (ms)	2.50/0.90	4.12/2.08	5.27/2.39	4.50/1.60
Flip angle (°)	20	20	10	10
Phase oversampling (%)	26	20	N/A	N/A
Slice oversampling (%)	20	0	N/A	N/A
Voxel size (mm^3^)	0.68 × 0.68 × 3.0	0.91 × 0.91 × 3.0	0.84 × 0.84 × 1.2	0.89 × 0.89 × 1.2
Temporal resolution (s)	5.2	4.3	120	120
Field of view (mm)	350	350	350	370
Fat suppression	None	None	SPAIR	SPAIR

**Table 2:** Lesion characteristics.

Characteristics	Value (Proportion)
Benign lesions	109 (0.63)
Adenosis	24 (0.14)
Fibroadenoma	19 (0.11)
Hyperplasia	6 (0.03)
Glandular tissue	4 (0.02)
Cyst	3 (0.02)
Inflammation	1 (0.01)
Other ^1^	51 (0.29)
Malignant lesions	64 (0.37)
Invasive ductal carcinoma	51 (0.29)
Invasive lobular carcinoma	4 (0.02)
Ductal carcinoma in situ	4 (0.02)
Micropapillary carcinoma	2 (0.01)
Apocrine carcinoma	1 (0.01)
Mucinous carcinoma	2 (0.01)
Lesion size (mm) ^2^	
Overall	19.9 ± 18.4
Malignant	28.6 ± 20.8
Benign	13.9 ± 13.6

^1^ The “Other” category included enhancement around fat necrosis, scar tissue, hyperplasia, atheroma cyst, regional background enhancement, and other benign-appearing enhancements not specified. ^2^ Data are ± standard deviation.

**Table 3:** Diagnostic performance of each model with ultrafast MRI under different threshold settings.

Threshold	2D CNN		LSTM		Combined	
	Sensitivity	Specificity	Sensitivity	Specificity	Sensitivity	Specificity
0.1	0.92 (0.91, 0.93)	0.25 (0.23, 0.27)	0.92 (0.91, 0.93)	0.22 (0.20, 0.24)	0.96 (0.95, 0.96)	0.14 (0.13, 0.16)
0.2	0.82 (0.81, 0.84)	0.56 (0.54, 0.58)	0.81 (0.80, 0.83)	0.48 (0.46, 0.50)	0.87 (0.86, 0.88)	0.48 (0.46, 0.50)
0.3	0.73 (0.72, 0.74)	0.76 (0.75, 0.77)	0.73 (0.72, 0.75)	0.65 (0.63, 0.67)	0.78 (0.77, 0.79)	0.72 (0.71, 0.74)
0.4	0.64 (0.63, 0.66)	0.88 (0.87, 0.88)	0.66 (0.64, 0.68)	0.78 (0.77, 0.79)	0.68 (0.67, 0.70)	0.86 (0.85, 0.86)
0.5	0.56 (0.55, 0.57)	0.93 (0.92, 0.93)	0.59 (0.57, 0.61)	0.88 (0.87, 0.88)	0.57 (0.55, 0.58)	0.92 (0.91, 0.93)
0.6	0.44 (0.43, 0.46)	0.95 (0.94, 0.95)	0.46 (0.44, 0.48)	0.92 (0.91, 0.93)	0.44 (0.43, 0.46)	0.96 (0.95, 0.96)
0.7	0.34 (0.33, 0.35)	0.96 (0.96, 0.97)	0.34 (0.33, 0.36)	0.94 (0.94, 0.95)	0.32 (0.31, 0.33)	0.98 (0.97, 0.98)
0.8	0.25 (0.23, 0.26)	0.98 (0.97, 0.98)	0.24 (0.22, 0.25)	0.97 (0.96, 0.97)	0.18 (0.17, 0.19)	0.99 (0.99, 0.99)
0.9	0.15 (0.14, 0.16)	0.99 (0.99, 0.99)	0.12 (0.10, 0.13)	0.99 (0.98, 0.99)	0.07 (0.06, 0.08)	1.0 N/A

## Data Availability

The data that support the findings of this study are available from the corresponding author upon reasonable request. The data are not publicly available due to privacy concerns, in accordance with GDPR.
